# Differentiated resistance training of the paravertebral muscles in patients with unstable spinal bone metastasis under concomitant radiotherapy: study protocol for a randomized pilot trial

**DOI:** 10.1186/s13063-017-1903-x

**Published:** 2017-03-31

**Authors:** Stefan Ezechiel Welte, Joachim Wiskemann, Friederike Scharhag-Rosenberger, Robert Förster, Tilman Bostel, Thomas Bruckner, Ingmar Schlampp, Eva Meyerhof, Tanja Sprave, Nils H. Nicolay, Jürgen Debus, Harald Rief

**Affiliations:** 1 Department of Radiation Oncology, University Hospital Heidelberg, Im Neuenheimer Feld 400, 69120 Heidelberg, Germany; 2 Department of Medical Biometry, University Hospital Heidelberg, Im Neuenheimer Feld 305, 69120 Heidelberg, Germany; 3 National Center for Radiation Oncology (NCRO), Heidelberg Institute for Radiation Oncology (HIRO), Im Neuenheimer Feld 400, 69120 Heidelberg, Germany; 4 Department of Medical Oncology, National Center for Tumor Diseases (NCT) Heidelberg, Heidelberg University Hospital, Heidelberg, Germany; 5 Department of Preventive Oncology, National Center for Tumor Diseases (NCT) Heidelberg, German Cancer Research Center (DKFZ), Heidelberg, Germany

**Keywords:** Bone metastases, Pathological fracture, Palliative radiotherapy

## Abstract

**Background:**

Metastatic bone disease is a common and severe complication in patients with advanced cancer. Radiotherapy (RT) has long been established as an effective local treatment for metastatic bone disorder. This study assesses the effects of RT combined with muscle-training exercises in patients with unstable bone metastases of the spinal column from solid tumors. The primary goal of this study is to evaluate the feasibility of muscle-training exercises concomitant to RT. Secondly, quality of life, fatigue, overall and bone survival, and local control will be assessed.

**Methods/Design:**

This study is a single-center, prospective, randomized, controlled, explorative intervention study with a parallel-group design to determine multidimensional effects of a course of exercises concomitant to RT on patients who have unstable metastases of the vertebral column, first under therapeutic instruction and subsequently performed by the patients themselves independently for strengthening the paravertebral muscles. On the days of radiation treatment the patients will be given four different types of exercises to ensure even isometric muscle training of all the spinal muscles. In the control group progressive muscle relaxation will be carried out parallel to RT. The patients will be randomized into two groups: differentiated muscle training or progressive muscle relaxation with 30 patients in each group.

**Discussion:**

Despite the clinical experience that RT is an effective treatment for bone metastases, there is insufficient evidence for a positive effect of the combination with muscle-training exercises in patients with unstable bone metastases. Our previous DISPO-1 trial showed that adding muscle-training exercises to RT is feasible, whereas this was not proven in patients with an unstable spinal column. Although associated with several methodological and practical challenges, this randomized controlled trial is needed.

**Trial registration:**

ClinicalTrials.gov, identifier: NCT02847754. Registered on 27 July 2016.

**Electronic supplementary material:**

The online version of this article (doi:10.1186/s13063-017-1903-x) contains supplementary material, which is available to authorized users.

## Background

Bone is the third most common organ affected by metastases, after the lung and liver [1]. Bone metastases of the spinal column are a common manifestation of distant relapse from many types of solid cancers, especially those arising in the lung, breast and prostate [2]. For hematological malignancies, bone involvement can also be extensive in patients with multiple myeloma, and bone may be a primary or secondary site of disease involvement in patients with lymphoma [1–4]. Thirty percent of all skeletal metastases and 10% of all primary bone tumors are located in the spinal column [5]. Spinal bone metastases are located in the lumbar (52%), thoracic (36%) and cervical (12%) regions [6]. The exact mechanism behind the occurrence of bone metastasis is not fully understood. It is postulated that bone metastases arise as a detachment of tumor cells from the primary tumor and are mainly dragged by intravascular penetration into the bone. In the bones, the tumor cells cause an imbalance in bone remodeling which leads to local changes of the bone structure presenting as osteolytic or mixed osteolytic/osteoblastic phenotype. Many metastatic bone lesions cause few or no symptoms but regarding skeletal-related events, pain is the most common symptom, and many patients with bone metastasis experience significant pain at some point in their disease course [7]. Other skeletal-related events can include limitations in daily activities, fatigue, pathological fractures, hypercalcemia and neurological deficits. Pathological fractures occur in 5% and spinal cord compression is found in 10–15% of patients [5]. As a part of the central axis, the spinal column is important for patients’ mobility. Impaired stability may lead to a devastating long-term impact on functioning, mobility, independence, health and quality of life (QoL).

We have already shown in our previous study (DISPO-1 trial) that in those patients with stable bone metastases of the spine concomitant radiotherapy (RT) and differentiated sports therapy are safe and feasible and have an advantageous effect on QoL [8–12]. This trial was designed to evaluate a differentiated strength training of the paravertebral muscles in cancer patients with unstable spinal bone metastasis under concomitant RT. Strengthening of the paravertebral musculature does not only have positive effects on the perception of pain, but may also improve QoL and fatigue. The implementation of isometric muscle training increases the blood flow in the affected vertebral segment. This circumstance may, in combination with RT, result in a better response to therapy. In patients with stable metastases there is consistent evidence that the risk of fracture in connection with an injury of the spinal cord involving neurological deficits is not increased [9]. Many patients develop significant anxiety from complications such as fracture, exacerbation of pain or neurological impairment. This fear of a serious event may create an unintended “vicious circle” consisting of immobility, pain and ever-decreasing physical performance. In almost all cases, pain, anxiety and impaired physical mobility are associated with a reduction in QoL and often lead to social negative effects. In our DISPO-1 study we showed that in combination with RT a targeted, regular and differentiated training of the paravertebral musculature in stable bone metastases is well tolerated by the majority of patients [9–12]. So far, however, no specific workout therapeutic measures in unstable bone metastases of the spine during and after RT have been described. The extent to which specific, regular and differentiated training of the spinal muscles can be performed may be diminished by the reduced general condition, the pain situation and the fear of fractures in affected patients. Therefore, the feasibility of this study is the greatest challenge. In a large retrospective study, we were able to show that abandoning general corset use in patients with spinal metastases does not significantly cause increased rates of pathological fractures [8]. The study is designed to integrate physical training with its multidimensional effects in patients with unstable bone metastases of the vertebrae who are undergoing RT. Therefore, the primary objective of this study is to evaluate the feasibility of an isometric training of the paravertebral musculature concomitant with palliative RT for unstable spinal bone metastases. Further study objectives are survival, treatment response to irradiation and clinical parameters such as pain, QoL and fatigue.

## Methods/Design

This is a single-centre, prospective, randomized, controlled, exploratory intervention study with a parallel-group design to determine the multidimensional effects of first, a guided, and thereafter, independently continued, targeted development training of the paravertebral muscles in patients with unstable spinal metastases concomitant to RT. Since the bone metastases of different patients may be located at different levels of the vertebral column, four different types of exercises are selected to ensure even isometric muscle training of the entire spinal musculature. To achieve exercises with high effectiveness, high quality and at the same time ensure high levels of safety, the study was designed and will be carried out by physical therapists, sports scientists as well as sports physicians and radiation oncologists in an interdisciplinary setting. In the control group a progressive muscle relaxation will be carried out parallel to RT. The plan foresees the recruitment, over a period of 12 months, of a total of 60 patients with unstable metastasis of the vertebral column who are scheduled to undergo RT. Prior to their enrollment into the study, the patients will receive a staging of the vertebral column in connection with their radiation treatment-planning computed tomography (CT) to measure bone density. The detection of unstable bone metastases is essential for accurate staging and optimal treatment. After the baseline results have been recorded, the patients will be randomized into one of the two groups: differentiated muscle training (*n* = 30) or progressive muscle relaxation (*n* = 30). The interventions will start at the same time as RT, taking place on each day of irradiation (Mondays to Fridays inclusive) for a period of 2 weeks. Each sport intervention as well as the progressive muscle relaxation will last approximately 15 min a day over the 2-week period. After completion of RT the training group will continue the exercise under the instruction of the therapist and later on their own independently at home. Patients will record the exercises and the pain load in a daily protocol report. Participants in the control group will do no further measurements at home. The target parameters will be measured and recorded at the end of the irradiation period (t_1_) and 12 weeks (t_2_) and 6 months following the end of the irradiation period (t_3_). Follow-up measurements are scheduled to take place 12, 18 and 24 months after the end of irradiation.

### Recruitment and randomization

Patients will be given information on the study by the medical personnel of the radiotherapy department in connection with the planning of the RT regimen. This will take place approximately 1–2 weeks prior to the start of RT. Importantly, the mentioned time range does not differ to the time range concerning regular patients treated outside the study.

A block randomization procedure will be used to ensure the even distribution of the patients into the two groups. The patients will then be assigned 1:1 into one of the two treatment arms by the study director (or an authorized representative) on the basis of the baseline measurements. The randomization procedure will be carried out by a central office. The study personnel responsible for the recruitment and baseline measurements will have no access to the randomization list, and the study director no influence on patient recruitment. The recruitment phase will be concluded with the attainment of the planned number of patients (60 patients in total). It will last for 12 months, and is scheduled to start in June 2017. Regular study participation will end 2 years after enrollment into the study or, where applicable, with the respective patient’s death.

### Inclusion criteria

- Patients with a histologically secured tumor diagnosis, with a secondarily diagnosed solitary/multiple metastatic processes in the thoracic or lumbar spine or in the os sacrum
- Indication for RT of the osseous metastatic processes
- Age: between 18 and 80 years
- Karnofsky Index ≥70 [13]
- Signed Declaration of Informed Consent
- Bisphosphonate therapy or anti-RANK ligand antibody therapy

### Exclusion criteria

- Significant neurological or psychiatric disorders, including dementia and epileptic seizures
- Other severe disorders that in the judgement of the study director may prevent the patient’s participation in the study
- Lacking or diminished legal capacity
- Any medical of psychological condition that the study director considers a preventive factor for the patient’s ability to complete the study or to adequately understand the scope of the study and to give their consent
- Unable to perform the exercises
- Indication for operation to the unstable spinal bone metastasis

### Intervention group

#### Sport concept: differentiated isometric exercise of the autochthonous muscles

Exercises in the “all-fours” position:

Starting position: arms and upper legs perpendicular to the floor, hands and knees positioned vertically under the shoulders and hips, respectively. The spine is as far as possible in the zero position over all segments. The elbows are slightly flexed. From the starting position, the right arm is anteverted at the shoulder from the sagittal level in the ventrocranial direction, at the very most until it is horizontal. The arm is then dropped again until it reaches the starting position, but it does not touch the ground again until the series of exercises is ended. The patient should keep their spine completely stable while the arm is being moved. The exercise is then repeated with the left arm. Duration: two series per arm, repeating each series of exercises ten times.

Exercises in the “swimming” position:

Starting position: prone, the toes are on the floor, the arms are stretched forward. The shoulder girdle and the arms are lifted from the ground and the view is directed toward the bottom. The exercise is executed by the arms, which move diametrically opposed in small, rapid movements up and down. The trunk is strained. Breathing should be continued during the exercise. If possible, the legs can be simultaneously lifted from the ground. Duration: two series each 30 s, with a 60-s break in between.

Exercises in the “forearm support” position:

Starting position: the body weight rests on the tiptoes and the forearms, elbows are placed directly under the shoulders. The body is tense, just raising the back, the buttocks are at a maximum of shoulder height and the face is directed to the ground. The position is to be held for 15 s and breathing continued here. Duration: two series, with a 60-s break in between.

Exercises with elastic “rubber” band:

Starting position: upright standing position, feet shoulder width apart. The knee joints are slightly bent and the buttocks and the torso tense. The elastic band is held with both arms at the level of the umbilicus. Duration: the elastic band is stretched until a slight tremor of the arms occurs, the trunk remains tense, while continuing breathing smoothly. The arms are moved slowly on the chest and back to the level of the umbilicus. During the entire range of motion power should be maintained. Duration: three series with 15-s duration intervals between series: 60–90 s.

### Control group

#### Physical measure: muscle relaxation

Study participants in the control arm will receive a progressive muscle relaxation instead of isometric muscle training. This will occur for about 2 weeks, parallel to the radiation treatment, carried out under guidance of an audio file. The sessions will last about 15 min. After completion of RT no further meetings are planned.

#### Questionnaire diagnostics

**Fig. 1:**
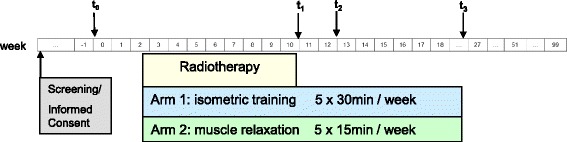
Flow chart of DISPO-2 trial; t_0_ = randomization, t_1_ = end of RT, t_2_ (3 months) = restaging, t_3_ (6 months) = restaging. *RT* radiotherapy

**Fig. 2:**
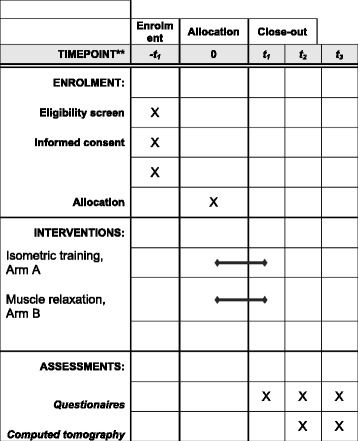
Standard Protocol Items: Recommendations for Interventional Trials (SPIRIT) figure

The secondary endpoints, such as fatigue, quality of life, and anxiety, will be recorded using validated questionnaires (EORTC QLQ C30 FA13 [14], EORTC QLQ C30 BM22 [15] and the questionnaire to record stress in cancer patients (FBK) according to Herschbach [16] (t_0_, t_2_, t_3_, Fig. 1). Furthermore, all patients will also be asked to record their pain history using a Pain Diary (documentation of medication daily during treatment, once weekly after the end of treatment, Visual Analogue Scale (VAS) pain scale). Figure 2 shows the Standard Protocol Items: Recommendations for Interventional Trials (SPIRIT) figure that guides our trial stages.

### Assessment of the primary and secondary endpoints

The aim of the study is to evaluate the feasibility of the defined training program. The feasibility as the primary endpoint was defined as the completion of the training program (mentioned above) up to 3 months after the end of RT (t_2_; Fig. 1) Additional file 1. Progression-free and fracture-free survival, improved response to RT by means of bone density, and clinical parameters, such as pain, quality of life, and fatigue, constitute secondary study objectives. In this study each fracture will be evaluated. Bone fracture rate will be calculated at baseline and after 3 and 6 months after RT by imaging, as well as if new clinical symptoms appear (Fig. 1).

Progression-free and fracture-free survival, improved response to RT by means of bone density, and clinical parameters, such as pain, quality of life, and fatigue, constitute secondary study objectives. In addition, the changes between baseline and week 12 and week 24 (end of intervention) regarding pain symptoms between the intervention arm (muscle exercises) and the control arm (progressive muscle relaxation) will be compared. The feasibility will be expressed in percent in tabular form and will cover the complete performance of the exercise program up to the t_2_ interval. As described above, the patients enrolled into the study will be subjected to a CT screening of the vertebral column with bone densitometry as per the standards of the follow-up investigation (t_2_). The study analyses provide for several follow-up meetings after the end of irradiation. Re-calcification should be the main outcome by means of increased bone density. Furthermore the psycho-oncological parameters on t_1_, t_2_ and t_3_ will be documented and evaluated. Following the RT period the patients will independently keep a Pain Diary and a record of their training exercises. No further radiological examinations will be conducted in the course of this study (Fig. 1).

### Radiotherapy

Treatment is simulated with a CT by using a 3-mm slice thickness taken within the involved vertebral region. On the basis of the planning CT, risk organs and clinical target volume (CTV) are contoured. The spinal cord is contoured on the basis of a visible target on the CT scan with the help of fusion with magnetic resonance imaging (MRI). CTV confirms planning target volume (PTV). The gross tumor volume (GTV) includes the macroscopic tumor. The PTV includes at least the metastasis-affected vertebra. PTV is equal to CTV. The total dose is also determined and varies between individual patients in individual irradiation formats. Consequently, there are also different single doses and fractions. Percutaneous RT occurs within 2–4 weeks using 6-MV individually formed beams (Linac, multileaf collimator) after CT scan-based 3D or intensity-modulated radiotherapy (IMRT) planning [17].

### Therapy dropout criteria

- At the patient’s wish
- Medical condition requiring the discontinuation of therapy in the opinion of the study director or patient
- Insufficient compliance

In case of early dropout from the study the planned follow-up scheme will be continued. For non-evaluable patients the reason in the Case Report Forms (CRF) is recorded. The evaluable patients have the opportunity to revoke individual investigations (for example, MRI) and still continue as a study patient.

### Statistical analysis

The total number of patients undergoing RT in the radiation oncology department of the Heidelberg University Clinic for metastatic processes in the vertebral column in the recruitment period is approximately 120, about 90 of whom will fulfill the inclusion criteria. The relatively weakly distinct compliance of this group of patients notwithstanding, it should be possible to achieve the planned recruitment target within a period of 6 months. Stratification factors were dispensed due to the expectation of collective patient homogeneity. On account of the explorative character of this study it is not possible to estimate the total number of cases; with a scheduled number of 30 patients per group, it will, however, be possible to detect a standardized mean-value effect [18] of 0.8 with a power of 80% and an *α* significance level of 5%.

## Discussion

Osseous metastases of the vertebral column are the main localization of bone metastases and are associated with a poor prognosis. Unstable bone-affecting metastatic processes in vertebral bodies constitute a frequent secondary disorder in connection with a variety of primary tumors. Palliative percutaneous RT is one of the options available on this occasion. On the one hand, symptoms, such as painful impairments of mobility, pain at rest, a fear of pathological fractures, and fatigue, result in a pronounced diminution in the patients’ quality of life, while on the other hand the therapy of such disorders involves protracted, cost- and time-intensive measures. Pathological fracture in patients with bone metastases is an acute risk with the danger of the emergence of symptoms of paraplegia. Because the development of such a fracture is so devastating to a cancer patient, increased emphasis is now being placed on prevention. To assess whether RT indeed improves unstable bone quality and bone strength in addition to differentiated sport therapy, we initiated this study. In line with our previous study (DISPO-1) the effect of RT on bone density showed that bone density increased after RT. However, there is insufficient evidence to conclude whether or not RT leads to improved bone quality and increased bone strength in patients suffering from unstable solid bone metastases. The aim of this explorative study is to evaluate the feasibility of muscle-training exercises and to evaluate the progression-free and fracture-free survival time and the improvement of unstable bone density, as well as to assess other clinical parameters, such as pain, quality of life, and fatigue, as secondary endpoints. A further objective of the study is to make a contribution to the integration of a regimen of physical training exercises with its multidimensional effects into future therapeutic concepts for patients with unstable osseous metastases of the vertebral bodies. No stratification factors will be used in this study. Additional complexity associated with the stratification procedure adds only little additional profit, and we believe that the randomization will provide balanced groups anyway. To the best of our knowledge, ours is the first study to compare the effects of physical training exercises in patients with an unstable spinal column. The long-term consequences of such interventions are currently unknown, and further research is necessary to expand on these findings.

### Trial status

Not completed patient recruitment.

## Additional file

Additional file 1: SPIRIT 2013 Checklist: recommended items to address in a clinical trial protocol and related documents. (DOC 121 kb)

## Abbreviations

CRF: Case Report Form
CT: Computed tomography
CTV: Clinical target volume
GCP: Good Clinical Practice
GTV: Gross tumor volume
PTV: Planning target volume
